# “Are CRT upgrade procedures more complex and associated with more complications than de novo CRT implantations?” A single centre experience

**DOI:** 10.1007/s12471-015-0771-9

**Published:** 2015-12-07

**Authors:** I.A.H. ter Horst, Y. Kuijpers, J. van ’t Sant, A.E. Tuinenburg, M.J. Cramer, M. Meine

**Affiliations:** 1 Department of Cardiology, University Medical Center Utrecht, Heidelberglaan 100, PO Box 85500, 3508 GA Utrecht, The Netherlands; 2 Facility of Medicine, Utrecht University, Utrecht, The Netherlands

**Keywords:** CRT, Upgrade, Complication

## Abstract

**Objective:**

The objective of the study was to examine whether cardiac resynchronisation therapy upgrade procedures are more complex and associated with more complications than de novo implantations.

**Method:**

We retrospectively compared 134 upgrade procedures performed between 2006–2012 with a random, equally sized, sample of de novo CRT device implantations in the same period. Procedural data and the occurrence of periprocedural (≤ 30 days) and long-term device-related (≤ 1 year) complications were analysed. Complications with consequences were defined as those in need of adjustment of standard care.

**Results:**

Median time to upgrade was 57 (31–115) months. There were no significant differences in procedure duration, radiation time or total hospitalisation between upgrades and de novo implantations. Perioperative complications occurred in 6.7 % of upgrade patients and in 9.0 % of de novo patients. The most frequently seen complications were phrenic nerve stimulation, coronary sinus dissection and pocket haematoma. Procedure success was comparable (upgrade: 98.5 % versus de novo: 96.3 %). A total of 236 patients completed 1 year of follow-up. Ten (4.2 %) patients had a long-term device-related complication with consequences including phrenic nerve stimulation, lead dislodgement/dysfunction, and infection (upgrade: 3.5 % versus de novo: 4.9 %).

**Conclusion:**

Upgrade procedures are not more complex nor associated with more complications than de novo CRT implantations.

## Introduction

Cardiac resynchronisation therapy (CRT) is most effective in heart failure patients with left bundle branch block as it specifically aims to resynchronise the delayed electrical activation of the left ventricle [1–2]. However, CRT is indicated in a much broader spectrum of patients with heart failure [3]. A large European survey among 141 hospitals demonstrated that approximately 25 % of CRT implantations are upgrade procedures [4]. Patients with an existing cardiac implantable electronic device (CIED) have a class I indication for upgrading to CRT when they experience a high percentage of ventricular pacing and remain in New York Heart Association (NYHA) class III to ambulatory IV with a left ventricular ejection fraction (LVEF) ≤ 35 %. Other reasons for upgrading to CRT include (i) worsening of heart failure (class) in patients with pre-existing wide QRS complexes, (ii) electrical remodelling due to heart failure or antiarrhythmic drug therapy with widening of the QRS complex, and (iii) evolving CRT guidelines.

Previous literature has shown that cardiac device replacement or upgrade procedures are associated with more complications and are more complex than de novo implantations due to the possibility of venous thrombosis and the risk of damaging or extracting old leads [5, 6]. The fear of a complicated procedure might prevent upgrading to CRT in patients with a clear indication. Moreover, reluctance about the need for an upgrade procedure in the future might make clinicians more willing to implant a de novo CRT device in heart failure patients with only a class IIb indication for CRT but a clear indication for a conventional (single or dual chamber) device.

We aimed to evaluate procedural data and periprocedural and long-term complication rates of upgrade procedures compared with de novo CRT implantations in our university medical centre.

## Method

### Study population, procedure and complication data

This retrospective study analysed data from all patients who had undergone an upgrade procedure from a single or dual chamber pacemaker or implantable cardioverter defibrillator (ICD) to a CRT device at the University Medical Centre of Utrecht (UMCU) in the period 2006–2012. An equally sized reference group of de novo implantations was randomly constructed out of patients implanted in the same period (*N* = 358). Procedures were performed by experienced operators, defined as performing at least 75 ICD/pacemaker implantations annually with at least one-third of these being CRT device implantations.

Procedural data as procedure length, radiation duration and hospitalisation duration were collected. Periprocedural complications (≤ 30 days) and long-term device-related complications (≤ 1 year) were divided into complications with and without consequences. Complications with consequences were defined as those in need of invasive procedures (e.g. lead revision/replacement, extraction of the system, pericardiocentesis) or any other action deviating from standard clinical care (prolonged hospitalisation, postponement of implant after start, transfer to the coronary or intensive care unit or the need to start anticoagulants).

### Statistical analysis

Baseline characteristics of the upgrade group were compared with the de novo implant group. Categorical variables are presented as percentages and the P-value was calculated using Pearson’s Chi-square test. Distribution of continuous variables was assessed by the Kolmogorov-Smirnov test for normality. If distribution was skewed, variables are presented as median with 25th to 75th percentile interquartile range (IQR) and the *P*-value was calculated by the Mann-Whitney U test. Normally distributed variables are presented as means with a standard deviation and a *P*-value calculated by the Student’s t test. The occurrence of specific periprocedural complications was compared between de novo and upgrade procedures using a Chi-square test. The hazard ratio for the risk of any periprocedural complication with consequences for the type of procedure (upgrade versus de novo implantation) was calculated by a crude logistic regression analysis. To identify any confounding effect of baseline characteristics on the risk of periprocedural complications after an upgrade procedure and de novo CRT implantation, multiple bivariate logistic regression analyses were performed, including procedure type and one baseline characteristic. The crude hazard ratio for procedure type and risk of periprocedural complications was adjusted for all baseline characteristic differences in a multivariable logistic regression analysis. The comparison of the occurrence of long-term complications by procedure type was performed by the Chi-square test. Patients with censored data due to loss to follow-up or death within 1 year of follow-up were excluded from this comparison.

## Results

### Patient characteristics of total population

This analysis included 134 patients who underwent an upgrade procedure to a CRT device and 134 randomly selected patients receiving a de novo CRT device. Of these 268 patients, 264 (98.5 %) received a CRT defibrillator (CRT-D), three (2.2 %) patients were upgraded to a CRT pacemaker (CRT-P) and one (0.7 %) patient received a de novo CRT-P. Fifty-four (40 %) patients where upgraded from a double chamber device and 81 (60 %) from a single chamber device. Seventy-six (57 %) upgrade patients were paced from the right ventricle at baseline. Upgrading took place 57 (31–115) months after implantation of the initial CIED.

Baseline data are presented in Table 1. Upgrade patients were more often males (*P* = 0.011) and of older age (*P* = 0.001) than the random sample of patients receiving a de novo CRT implantation. They more often had ischaemic cardiomyopathy (*P* = 0.049) and a history of atrial fibrillation (*P* = 0.008) and were more often on amiodarone (*P* < 0.001) and a vitamin K antagonist (*P* = 0.022). No baseline characteristic had a significant confounding effect on the hazard ratio for procedure type and the risk of periprocedural complications.

**Table 1:** Baseline comparison between patients receiving an upgrade to a CRT device and those receiving a de novo CRT device

Baseline characteristics	Total population (*N* = 268)	Upgrade(*N* = 134)	De novo implants (*N* = 134)	*P-value*
Age *(years)*	69(61–74)	71(63–75)	67(60–72)	**0.001**
Male (N(%))	202(± 75)	110(± 82)	92(± 69)	**0.011**
BMI	26(24–29)	26(24–29)	26(24–30)	0.450
**NYHA class** (*N*, %)^a^				0.080
I	2(1)	1(1)	1(1)	
II	36(13)	13(10)	23(17)	
III	213(80)	110(82)	103(77)	
IV	17(6)	10(8)	7(5)	
Ischaemic CMP *(N, %)*	148(55)	82(61)	66(49)	**0.049**
LVEF (%)	23( ± 7)	24( ± 7)	23( ± 7)	0.239
BNP	141(68–262)	146(73–273)	130(50–226)	0.112
Atrium fibrillation *(N, %)*	60(22)	39(29)	21(16)	**0.008**
**Cardiac medication** *(N, %)*				
Diuretic	231(88)	117(88)	114(87)	0.816
Beta-blocking	201(76)	100(76)	101(76)	0.940
ACE inhibiting	227(86)	110(83)	117(89)	0.158
**Other medication**				
Amiodarone	54(21)	39(29)	15(11)	< **0.001**
**Use of anticoagulation** *(N, %)*				
Vitamin-K antagonist	191(72)	104(78)	87(65)	**0.022**
Other	57(21)	23(17)	34(25)	0.101
INR *(N = 191)*	1.7(1.3–2.1)	1.7(1.4–2.1)	1.5(1.3–1.9)	0.053
**Comorbidity**				
Diabetes mellitus *(N, %)*	62(23)	29(22)	33(25)	0.540
COPD *(N, %)*	34(13)	17(13)	17(13)	0.981
Renal function (eGFR)	57(19)	55(20)	59(19)	0.090
**Subclavian/anonyma vein thrombosis** *(N, %)*	11(4)	11(8)	0(0)	**0.001**
**Time since initial CIED implantation** *(months)*		57(31–115)	–	–

± Standard deviation deviation, range; 25th to 75th percentile inter quartile range.

*ACE* angiotensin I converting enzyme, *BMI* body mass index, *BNP* brain natriuretic peptide, *CIED* cardiac implantable electronic device, *CMP* cardiomyopathy, *COPD* chronic obstructive pulmonary disease, *INR* international normalised ratio, *LVEF* left ventricular ejection fraction, *NYHA* New York Heart Association, *SD* standard deviation.

^a^NYHA III and IV versus I, II

### Procedure

Procedural data are presented in Table 2. Seventy-three (27 %) procedures were extended by study-related LV pressure (LV dP/dt max) measurements to optimise lead positions and device settings. Standard procedures had a median length of 139 (69) minutes. Median radiation time was 25 (20) minutes and median hospitalisation after implantation, including rehospitalisation for another attempt to complete CRT implantation after initial LV lead placement failure, was 2 (2) days. There were no significant differences in these procedural data between upgrade and de novo CRT implantation procedures. At baseline, ten (8 %) patients undergoing an upgrade procedure had a subclavian (or anonyma) vein thrombosis. In six of these patients the vein was recanalised, in two a contralateral implant was chosen and in two patients the occlusion could be passed. In 124 (92 %) de novo procedures the intention was to implant three leads. Ten (8 %) patients did not receive a right atrial lead because of permanent atrial fibrillation. Of the upgrade patients 91 (68 %) received two new leads, three leads were placed in 16 (12 %) patients and 27 (20 %) patients only received an additional LV lead.

**Table 2:** Procedural data

Procedural data (median, IQR)	Total	Upgrade	De novo	*P*-value
Length procedure (min)	156(78)	145(71)	163(81)	0.062
Standard procedure (*N* = 194)	139(69)	135(75)	142(61)	0.515
Procedure with LV dP/dt max measurements^a^	187(66)	171(72)	195(42)	0.176
Radiation time (min)	25(20)	25(19)	25(21)	0.595
Total hospitalisation time^b^ (days)	2(2)	1(2)	2(2)	0.418
**Leads added or implanted** (intention to) (*N*, %)				< 0.001
1		27(20 %)	0(0)	
2		91(68 %)	10(8 %)	
3		16(12 %)	124(92 %)	
**N of operating specialists** ^**c**^				1.000
*N = 1* (experienced cardiologist)	224(85)	112(85)	112(85)	
*N = 2* (fellow with experienced cardiologist or two experienced cardiologists)	40(15)	20(15)	20(15)	

^a^of which 39.7 % upgrade procedures.

^b^starting after implantation including additional hospitalisation for later LV lead placement after initial failure or re-hospitalisation due to implantation or device related complications.

^c^4 patients had missing data on implanting physician.

### Periprocedural complications

The periprocedural complication rate is presented in Table 3. In total 21 (7.8 %) patients experienced a complication with consequences. There were no significant differences between the upgrade and the de novo group. The crude hazard ratio for periprocedural complications (with consequences) for upgrade procedures compared with de novo CRT implantations was 1.692 (*P* = 0.260) and when adjusted for baseline differences between the two groups it was 1.779 (*P* = 0.249) (Table 4). One patient developed acute decreased saturation during an upgrade procedure probably based on cardiac asthma. Saturation did not improve with pharmacological treatment or intubation, and resuscitation was required, which the patient did not survive. In seven (2.6 %) patients transvenous LV lead placement was unsuccessful due to difficulty in visualising or cannulating the coronary sinus. Phrenic nerve stimulation was seen in 14 (5.3 %) patients and usually managed by adjustments in the pacing configuration, but in one case lead revision was needed shortly after implantation. Coronary sinus dissection (3 %) did not have any invasive consequences. Three (1.1 %) patients had a pneumothorax in need of drainage. Pocket haematoma was seen in 12 (4.5 %) of the total 268 patients; of these four upgrade patients needed to be re-hospitalised or needed blood transfusion.

**Table 3:** Periprocedural complications

Complication (*N*, %)	Total (*N* = 268)	Upgrade (*N* = 134)	De novo (*N* = 134)
**Perforation/dissection entrance vein**	2(0.7)	1(0.8)	1(0.8)
**CS dissection**			
Conservative	8(3.0)	4(3.0)	4(3.0)
With consequences	–	–	–
**Cardiac tamponade**			
Conservative	1(0.4)	–	1(0.8)
Pericardiocentesis	1(0.4)	–	1(0.8)
**Cardiac arrest during implant** ^b^	1(0.4)	–	1(0.8)
**Haemorrhage during**			
**implant**	2(0.7)	2(1.5)	–
Conservative	1(0.4)	–	1(0.8)
Transfer to CCU			
**LV lead placement failure (transvenous)**	7(2.6)	2(1.5)	5(3.7)
**Pneumothorax**			
Conservative	2(0.7)	–	2(1.5)
Drainage	3(1.1)	1(0.8)	2(1.5)
**Pocket haematoma**			
Conservative	8(3.0)	4(3.0)	4(3.0)
Re-hospitalisation/transfusion^d^	4(1.5)	4(3.0)	0(0)
**PNS**			
Conservative	13(4.9)	4(3.0)	9(6.7)^e^
Lead revision	1(0.4)	–	1(0.8)
**Thrombosis v. subclavian post implant** ^c^	1(0.4)	1(0.4)	–
**Death (implant related)**	1(0.4)	1(0.8)	–
**Total**	56(20.9)	24(17.9)	32(23.9)
**With consequences**	21(7.8)	9(6.7)	12(9.0)

*CCU* coronary care unit, *CS* coronary sinus, *LV* left ventricular, *PNS* phrenic nerve stimulation.

^a^leading to postponement of implant or conversion to another entrance vein.

^b^due to temporary AV block without an escape rhythm for which a short period of resuscitation followed by temporary pacing by the ICD lead after which recovery of conduction.

^c^for which compression stockings and anticoagulation.

^d^There was no need for re-intervention.

^e^ *P* < 0.05 for comparison between de novo and upgrade procedures.

**Table 4:**
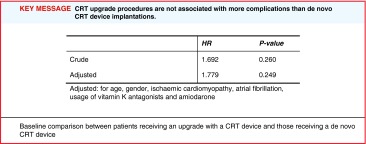
Hazard ratio for upgrade versus de novo CRT implantation procedures and the risk of periprocedural complications, adjusted for differences in baseline characteristics between upgrade and de novo patients.

### Long-term follow-up

Long-term device-related complications are listed in Table 5. A total of 236 patients completed 1 year of follow-up. Ten (4.2 %) patients had a long-term device-related complication with consequences; there was no difference between upgrade (3.5 %) and de novo (4.9 %) patients. Lead dysfunction in need of lead replacement occurred once in a de novo and an upgrade patient. Lead dislodgment in need of lead revision was seen in three de novo patients and in one upgrade patient. Device-related endocarditis requiring extraction of the system was seen once in both a de novo and in an upgrade patient.

**Table 5:** Long-term device-related complications during 1 year of follow-up

Complication (*N*, %)	Total *N* = 236^a^	Upgrade *N* = 113	De novo *N* = 123
**PNS**			
Conservative	11(4.7)	6(5.3)	5(4.1)
Lead revision	1(0.4)	1(0.9)	–
**Lead dysfunction**			
Conservative	6(2.5)	3(2.7)	3(2.4)
Lead replacement	2(0.8)	1(0.9)	1(0.8)
**Lead dislodgement**			
Conservative	4(1.7)	1(0.9)	3(2.4)
Lead revision	4(1.7)	1(0.9)	3(2.4)
**Infection**			
Conservative	2(0.8)	-	2(1.6)
AB/hospitalisation	1(0.4)	-	1(0.8)
Extraction	2(0.8)	1(0.9)	1(0.8)
**Total**	33(14.0)	14(12.4)	19(15.4)
**With consequences**	10(4.2)	4(3.5)	6(4.9)
Loss to FU	5	1	4
Htx	3	3	–
Death^b^	27	19	8
N before death or Htx long-term complication	*3*	*2*	*1*

*AB* antibiotics, *Htx* heart transplantation, *FU* follow-up, *PNS* phrenic nerve stimulation.

^a^excluded were those patients who were loss to follow up (*N*=5) or died/received an Htx during follow up before any long term complication could occur (*N* = 32).

^b^One upgrade patient died during procedure.

† *P* < 0.05 for comparison between de novo and upgrade procedures.

## Discussion

Our single-centre experience did not find any significant differences in either procedure length, radiation duration, total hospitalisation, or in periprocedural and long-term device-related complication rates when comparing upgrade procedures with de novo CRT implantations.

Previously, the RAFT study showed that CRT-D reduced rates of death and hospitalisation for heart failure when compared with ICD therapy only. However, device-related hospitalisation was significantly higher for the CRT-D group compared with the ICD-only group [7]. The increased risk of complications of a triple chamber device compared with conventional single and dual chamber pacemakers or ICDs was also highlighted by a large nationwide cohort of 5918 Danish patients who underwent a CIED implantation, in which patients receiving a CRT device showed the highest complication rate [8]. Therefore, the decision to implant a triple chamber device needs to be carefully considered.

For patients with a class IIb indication for CRT but a clear indication for a conventional ICD the alternative to a de novo CRT implantation is to await the potential need for an upgrade to CRT. However, as was shown by Poole et al., CRT upgrade is a high-risk procedure as well, with an 18.7 % risk of any complication during a follow-up of six months [5]. The fear of complications associated with an upgrade procedure might prevent the upgrade to CRT in patients with a clear indication. This was suggested by the results of Scott et al. who performed a single-centre retrospective observational study evaluating the implantation of ICDs for a period of 5 years. In this period 549 new ICDs were implanted of which 73 % single or dual chamber ICDs and 27 % CRT-D. When they applied alternative indication criteria (LVEF ≤ 30 %, QRS ≥ 130 ms, NYHA I–IV), 42.6 % of the ICD recipients met the criteria for CRT at initial implant. However, the upgrade rate to CRT at 5 years was only 5.1 % [9].

We showed a comparable complication rate between upgrade and de novo CRT implantation procedures. This is in line with the results of the European Survey which showed that there were no significant differences in complication rates when comparing 692 upgrades with 1675 de novo CRT procedures at 141 centres in Europe [4]. However, this was a randomised trial and such trials commonly report less complications than in a real-life setting due to more strict patient selection and more experienced operators. On the contrary, the previously mentioned Danish population-based cohort of CIED implantations, including 445 CRT-D implantations, showed that CRT upgrade procedures or lead revisions were associated with more complications than CRT de novo implantations [8]. As both our study and the Danish cohort study reflect the results of a real-life setting the differences are not likely to be based on patient selection. In our retrospective study, patient selection was equal for both de novo and upgrade procedures in that it followed standard clinical care in which the benefit of CRT is balanced against the risk of complications. It might be that the operators in our tertiary centre were more experienced in implanting CRT devices. Although most procedures in the Danish cohort were also performed by operators with an annual volume of at least 75 ICD or pacemaker implantations, it is not clear whether the experience in implanting a CRT device was as high as in our centre [8]. Another point of concern for many clinicians is the fear of a more complex procedure when a single or dual chamber pacemaker or ICD needs to be upgraded, compared with a de novo implantation of a CRT device, due to the possibility of damaging or extracting old leads. Four upgrade patients experienced lead dysfunction after the procedure, which is comparable with numbers in previous literature [5, 10]. These were three old leads (two right atrial leads and one right ventricular lead) and one newly placed right atrial lead. The dysfunction of the old leads was seen 2–12 months after upgrade procedure. Moreover, this was done due to damage to the old lead during the procedure in only one of 16 upgrade patients who received a completely new system. Therefore, our data seem to indicate that when an upgrade procedure is performed by an experienced operator the risk of damaging old leads is small. Furthermore, we found no significant difference in procedure duration comparing upgrade procedures with de novo implantations, but there was a slight trend towards an even shorter procedure length for upgrade procedures. This suggests comparable complexity as one would expect the upgrade procedure to require a smaller amount of time, as in most upgrades less leads are added than implanted during a de novo implantation. Complicating factors such as subclavian vein thrombosis only slightly increased the median procedure duration compared with standard procedures (159 (95) minutes versus 139 (69) minutes). Moreover, in our upgrade group the procedure success was 98.5 % and did not significantly differ for the randomly selected de novo CRT implantation procedures. A meta-analysis of 9082 patients in 25 CRT trials showed an implantation success rate of 94.4 % indicating that the success rate of upgrade procedures was most definitely not lower compared with de novo procedures [11].

## Clinical implication and future directions

Decisions on device type (conventional or triple chamber pacemaker or ICD) can be difficult in patients with an indication for a conventional pacemaker or ICD without a clear class I or IIa indication for CRT. Based on fulfilment of indication criteria for CRT the chance of benefit from CRT in these patients is low and the complication rate increases with the number of leads implanted. However, when a conventional pacemaker or ICD is opted for the need for upgrading can eventually arise. This means the patient is exposed to two procedures, where it is generally believed that an upgrade procedure is more complex and associated with a higher complication rate. This single-centre experience of a Dutch university hospital shows neither a higher complexity nor a higher complication rate of upgrade procedures to CRT compared with de novo CRT implantations. Therefore, CRT upgrade procedures should be performed when indicated without fear for a higher complication rate. However, as upgrading means an additional procedure is needed, future research should focus on identifying patient characteristics that predict the necessity for upgrading to CRT, especially for those patients in need of upgrading within the life expectancy of the initial device.

## Limitations

Due to the retrospective design of the study only complications properly documented in the electronic patient file were identified and not all baseline data were available for all patients. However, the advantage of the retrospective design is that it reflects a real-life setting of these device implantations. Furthermore, although none of the baseline characteristics showed a confounding effect on the risk of periprocedural complications, it is possible that some data that were not collected confounded the results, for instance steroid use. Finally, as this study shows the results of a single-centre experience, the results can only be applicable for a comparable centre.

## Conclusion

In a device implantation centre with experienced operators, CRT upgrade procedures are not associated with more complications nor do they seem more complex compared with de novo CRT implantations.

### Funding

None.

### Conflict of interests

None declared.
